# Fenugreek Is Superior to Guar Gum, Locust Bean Gum and Tara Gum for Metabolic Health: A Systematic Review

**DOI:** 10.3390/nu18142394

**Published:** 2026-07-22

**Authors:** Anna Evelin Juhász, Tímea Klaudia Kara, Éva Csajbókné Csobod, Réka Juhász

**Affiliations:** Department of Dietetics and Nutrition Science, Semmelweis University, Vas Str. 17, 1088 Budapest, Hungary; juhasz.anna.evelin@semmelweis.hu (A.E.J.); kara.timea@gmail.com (T.K.K.); csajbokne.csobod.eva@semmelweis.hu (É.C.C.)

**Keywords:** galactomannan, guar gum, fenugreek, dietary fiber, glycemic control, lipid profile, systematic review

## Abstract

**Background**: Guar (*Cyamopsis tetragonoloba*), locust bean (*Ceratonia siliqua*), fenugreek (*Trigonella foenum-graecum*), and tara (*Caesalpinia spinosa*) are plant-derived materials that contain galactomannans as their major polysaccharide component. Galactomannans are soluble, highly viscous, and fermentable dietary fibers widely used in the food, pharmaceutical, and cosmetic industries because of their favorable technological properties. **Methods**: We performed our systematic search on 16 April 2026, in MEDLINE (via PubMed), Scopus, and the Cochrane Central Register of Controlled Trials (CENTRAL), without language restrictions. Eligible randomized controlled trials compared the effects of guar gum, locust bean gum, and fenugreek on glycemic parameters, lipid profile, and body weight-related outcomes in healthy adults or patients with metabolic disorders. **Results**: A total of 50 randomized controlled trials were included in the analysis. Of these, 27 investigated guar gum, 20 fenugreek (12 seed powder, 7 seed extract, 1 leaf extract), two mixed galactomannan preparations, and one locust bean gum intervention. Considerable heterogeneity was observed across studies regarding participant characteristics, intervention doses, and treatment duration. Nevertheless, fenugreek-derived preparations consistently improved glycemic outcomes. In contrast, guar gum demonstrated more consistent effects on lipid parameters. Effects on body weight and body mass index were less consistent across studies. **Conclusions**: Our findings suggest that the metabolic effects of galactomannans are source-dependent, with fenugreek-based interventions appearing to have greater potential for improving glycemic outcomes, while guar gum may be more effective in improving lipid parameters. Since these fibers can be easily consumed in the form of dietary supplements, it is worth considering their regular, daily intake. The protocol was prospectively registered in the PROSPERO database (CRD420261368159).

## 1. Introduction

Galactomannans are naturally occurring plant polysaccharides found primarily in the endosperm of leguminous plant seeds. The main natural sources of galactomannans include guar (*Cyamopsis tetragonoloba*), locust bean/carob seed *(Ceratonia siliqua*), fenugreek (*Trigonella foenum-graecum*), and tara gum flour (*Caesalpinia spinosa*). Their chemical structure consists of a linear backbone composed of mannose units linked by β-(1→4)-glycosidic bonds, to which galactose side chains are attached at varying intervals via α-(1→6)-glycosidic bonds. Due to this molecular structure, galactomannans are resistant to digestion by human digestive enzymes. Their physical properties are largely determined by the mannose-to-galactose ratio: the higher the galactose content, the greater their water solubility. In addition, this ratio influences their viscosity, gel-forming ability, and interactions with other polymers. Accordingly, galactomannans are classified as soluble, highly viscous, and fermentable dietary fibers. Due to their favorable technological properties, they are widely used in the food, pharmaceutical, and cosmetic industries as thickening, stabilizing, emulsifying, and gelling agents [1]. Guar gum and fenugreek are produced on a large industrial scale, with India serving as the world’s leading producer and exporter, supplying several hundred thousand tons annually to international markets. In contrast, locust bean gum and tara gum are manufactured on a considerably smaller scale. Their availability in powdered form also facilitates precise dosing and practical use as dietary supplements. In the European Union, guar gum (E412), locust bean gum (E410), and tara gum (E417) are approved food additives with assigned E numbers, highlighting their established safety and broad applicability in food technology [2]. Among all galactomannan sources, guar gum, locust bean gum, and fenugreek gum have been studied the most due to their widespread use as food thickeners and dietary fibers [1].

Guar gum is extracted from the seeds of the leguminous plant *Cyamopsis tetragonoloba*, which is cultivated primarily on the Indian subcontinent. Its industrial production began in the 1950s, mainly as a substitute for locust bean gum due to its lower cost and greater availability. Today, India is the world’s largest producer of guar gum, accounting for approximately 80% of global production. In the food industry, it is primarily used as a thickening and stabilizing agent, as well as to inhibit ice crystal formation in frozen products and ice cream [3].

Locust bean gum is a natural polymer extracted from the seeds of *Ceratonia siliqua*, accounting for approximately one third of the seed’s total mass. In the food industry, it is approved for use as a thickening, stabilizing, emulsifying, and gelling agent. On its own, it is not considered an effective gelling agent; rather, its functionality is enhanced when combined with other gums. Since its production is less economical than that of guar gum, it is mainly used in applications where guar gum has certain limitations, such as gel formation in combination with other substances, frozen foods, and cheese, yogurt, and sour cream products [4].

Fenugreek is obtained from the seeds of *Trigonella foenum-graecum*, a plant native to Southern Europe and Western Asia. Its industrial production began in the 1990s, and its production has been steadily increasing ever since. Among galactomannans, fenugreek is the most water-soluble and has the highest galactose content (approximately 48%). However, its viscosity-increasing capacity is lower than that of guar gum and locust bean gum, and it is not considered a gelling gum, even when combined with other polysaccharides. In terms of application, fenugreek is used primarily as a culinary spice and medicinal plant rather than as an industrial food additive [5].

Based on the above, galactomannans can be considered widely available, safe, and well-utilizable dietary fibers [6]. In addition to their favorable technological and physicochemical properties, numerous studies have also reported their potential health benefits. Our previous network meta-analysis, which included 46 randomized controlled trials and compared 16 different soluble fibers in patients with type 2 diabetes, identified galactomannan derived from fenugreek as one of the most effective fibers, showing significantly greater improvements in HbA1c, fasting blood glucose, serum triglycerides, and LDL cholesterol levels compared with several other fiber types [7]. An earlier meta-analysis that focused on fenugreek supplementation also demonstrated significant reductions in fasting and postprandial glucose levels, although effects on lipid parameters were less consistent [8]. In addition to fenugreek, guar gum has also been extensively investigated. Recent meta-analyses have shown that guar gum supplementation improves lipid parameters, particularly total and LDL cholesterol levels, although its effects on triglycerides, HDL cholesterol, and body weight appear to be less consistent and may depend on dose and population characteristics [9,10].

Despite these promising findings, the overall evidence remains heterogeneous, with substantial variability in study design, intervention characteristics, and reported outcomes. Therefore, a comprehensive synthesis of the available evidence is needed to better understand the effects of galactomannans on key metabolic outcomes. The aim of this systematic review is to investigate the effects of different galactomannan sources on glycemic parameters, lipid profile, and body weight-related outcomes in both healthy individuals and those with obesity or metabolic disorders.

## 2. Materials and Methods

This systematic review was conducted and reported in accordance with the PRISMA 2020 statement [11]. The protocol was prospectively registered in the PROSPERO database (CRD420261368159) and adhered to throughout the study.

### 2.1. Eligibility Criteria

The inclusion criteria comprised randomized controlled trials (RCTs) conducted in adults (>18 years), including both healthy individuals and those with obesity or metabolic disorders, which evaluated the effects of galactomannan supplementation compared with a control condition (e.g., placebo, no intervention, or alternative dietary intervention). Eligible studies were required to report at least one of the following outcomes: glycemic parameters (e.g., HbA1c, fasting blood glucose, fasting insulin, postprandial glucose, glucose area under the curve (AUC) or HOMA-IR), lipid profile (e.g., total cholesterol, LDL, HDL, or triglycerides), or body weight-related outcomes (e.g., body weight or body mass index). Studies involving children, pregnant women, or lactating women were excluded. In addition, studies including participants with tumors, inflammatory bowel disease (IBD), or gastroesophageal reflux disease (GERD) were also excluded.

### 2.2. Information Sources

Our systematic search was conducted on 16 April 2026, in 3 scientific databases: MEDLINE (via PubMed), Scopus, and Cochrane Central Register of Controlled Trials (CENTRAL). No language or other filters were applied.

### 2.3. Search Strategy

During the systematic search, the main concepts were galactomannan sources (e.g., guar gum, locust bean gum, fenugreek), metabolic outcomes (e.g., glycemic and lipid parameters, body weight or body mass index), and randomized controlled trials. The full search key was ((galactomannan) OR (galactomannans) OR (fenugreek) OR (trigonella foenum-graecum) OR (carob) OR (Ceratonia siliqua) or (guar gum) OR (guaran) OR (Cyamopsis tetragonoloba) OR (tara gum)) AND ((glycemic parameters) OR (hgba1c) OR (hemoglobin A1C) OR (FBG) OR (fasting blood glucose) OR (FI) OR (Fasting insuline) OR (HOMA IR) OR (2-h postprandial glucose) OR (two-hour postprandial glucose) OR (lipid parameters) OR (LDL-Cholesterol) OR (HDL-cholesterol) OR (total cholesterol) OR (triglyceride) OR (HDL) OR (LDL) OR (obesity) OR (overweight) OR (weight control) OR (weight loss) OR (BMI) OR (body mass index)).

### 2.4. Selection Process

All search results were imported into EndNote (Clarivate Analytics) for citation management, and duplicates were removed prior to screening. Two authors (A.E.J. and T.K.K.) independently screened titles, abstracts, and full texts according to predefined eligibility criteria, with disagreements resolved by consensus or by a third review author (R.J.).

### 2.5. Data Collection

Two authors (A.E.J. and T.K.K.) independently extracted data from the included studies using a standardized data extraction form. Discrepancies were resolved by consensus or by consultation with a third review author (R.J.). The following data were extracted: study characteristics (first author, year of publication, country, study design, and duration), participant characteristics (sample size, age, sex), details of the intervention and control (type and dose of galactomannan), and relevant outcomes (glycemic parameters, lipid profile, and body weight-related outcomes). When necessary, corresponding authors were contacted to obtain missing data.

### 2.6. Quality Assessment

Risk of bias was assessed independently by two authors (A.E.J. and T.K.K.) using version 2 of the Cochrane Risk of Bias tool for randomized trials (RoB 2) for all outcomes [12]. The following domains were evaluated: the randomization process, deviations from intended interventions, missing outcome data, measurement of the outcome, and selection of the reported results. Each domain was judged as having low risk of bias, some concerns, or high risk of bias. Discrepancies between authors were resolved by consensus or by consultation with a third review author (R.J.).

### 2.7. Data Synthesis

The included studies were categorized based on the primary outcome domains, specifically glycemic parameters, lipid profile, and body weight-related outcomes. Findings were summarized descriptively, considering the direction, extent, and statistical significance of the reported effects in individual studies. Owing to the heterogeneity in study design, intervention characteristics, and outcome assessments, as well as the limited number of studies suitable for direct comparison within each outcome domain, a meta-analysis was deemed inappropriate.

## 3. Results

### 3.1. Search and Selection

**Figure 1:**
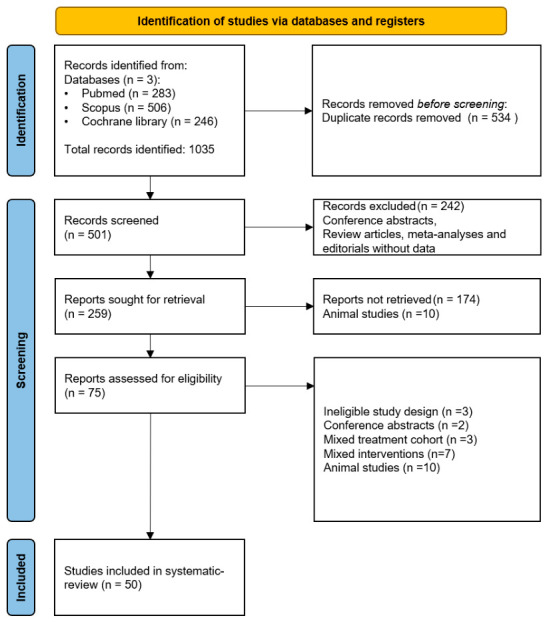
PRISMA 2020 flowchart representing the study selection process.

Altogether, 1035 studies were identified by our systematic search. After duplicate records were removed and the remaining records were selected, we identified 50 eligible studies for qualitative synthesis. The selection process is shown in Figure 1.

### 3.2. Main Characteristics of Included Studies

A total of 50 randomized controlled trials were included in the analysis. Of these, 27 investigated guar gum, 20 fenugreek (12 seed powder, 7 seed extract, 1 leaf extract), two galactomannan, and one locust bean gum intervention. The administered doses varied widely, ranging from 50 mg to 100 g per day. Specifically, fenugreek doses ranged from 50 mg to 100 g, galactomannan from 1 to 16 g, guar gum from 2 to 40 g, and locust bean gum was administered at a dose of 6 g. The duration of interventions ranged from acute postprandial studies (2 h) to long-term trials lasting up to 12 months, although most studies were conducted over a period of 6–8 weeks. The studies were conducted across a wide range of geographical regions, with the majority originating from India, North America (USA and Canada), and European countries including the United Kingdom, Finland, and the Netherlands. The study populations were heterogeneous, including individuals with type 2 diabetes, overweight or obesity, hyperlipidemia, as well as healthy participants. No major harmful side effects of any fiber type were reported in any of the included studies. The baseline characteristics of the included articles are detailed in Table 1.

### 3.3. Glycemic Levels

Among the 40 studies evaluating glycemic parameters, the majority focused on guar gum (*n* = 19) and fenugreek (*n* = 18), whereas only a small number of studies investigated galactomannan (*n* = 2) and locust bean gum (*n* = 1). Glycemic outcomes assessed across these studies included fasting blood glucose (FBG), postprandial glucose response (PPG), glucose area under the curve (AUC), and glycated hemoglobin (HbA1c). The most pronounced improvements in glycemic parameters varied across outcome measures. The largest reduction in FBG was reported by Rehman et al., where FBG decreased from 157.33 mg/dL to 108.2 mg/dL following fenugreek seed powder supplementation at a dose of 20 g/day for 2 months in patients with type 2 diabetes [49]. In terms of postprandial glycemic response, Robert al. observed the greatest decrease in glucose AUC, with a reduction of 39.2% after consumption of fenugreek seed powder-enriched products in a 2 h acute study conducted in healthy individuals [51]. The most substantial improvement in long-term glycemic control was reported by Hota et al., where HbA1c levels decreased from 8.1 ± 1.8% to 5.2 ± 3.6% after 12 weeks of fenugreek seed extract supplementation at a dose of 1 g/day in patients with type 2 diabetes. However, the authors noted that this reduction was not statistically significant when compared to the placebo group [30]. 

**Figure 2:**
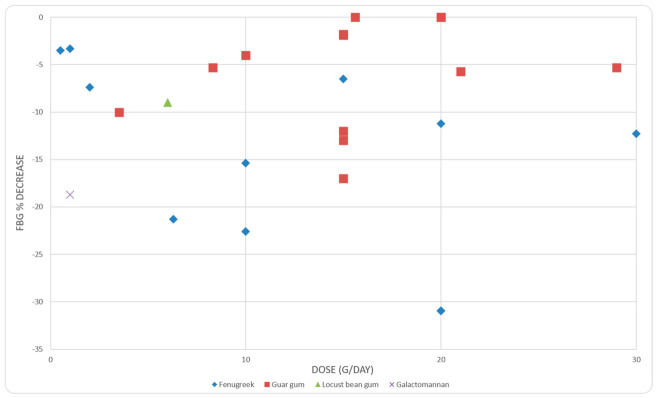
The relationship between fiber dose and the percentage change in FBG across the included studies [15,16,20,21,23,25,26,27,30,34,35,36,38,41,42,43,44,46,48,49,50,55,58,59,61]. Each point represents an individual study categorized by fiber type.

Given the absence of a clear dose–response relationship for FBG, a separate figure was created to allow a more detailed visual exploration of the dose-dependent effects (Figure 2). Reductions ranged from minimal changes close to 0% to decreases exceeding 31.2%. The largest decreases were primarily reported in studies using fenugreek preparations, while guar gum interventions generally showed more moderate effects. Locust bean gum and galactomannan were represented by only one study, limiting direct comparison. One study administering 100 g/day fenugreek was excluded from the figure because its exceptionally high dose substantially distorted the scale and reduced the interpretability of the graphical presentation [52]. No clear dose–response relationship was observed, as similar reductions in FBG were reported across a broad range of doses. Both low and high doses were associated with variable reductions in fasting blood glucose levels.

### 3.4. Lipid Levels

Lipid-related outcomes were evaluated in 31 of the 50 included studies, including total cholesterol (TC), low-density lipoprotein cholesterol (LDL), high-density lipoprotein cholesterol (HDL), and triglycerides (TGs). The majority of these studies investigated guar gum (*n* = 21), followed by fenugreek (*n* = 8), while one study each examined galactomannan and locust bean gum.

**Figure 3:**
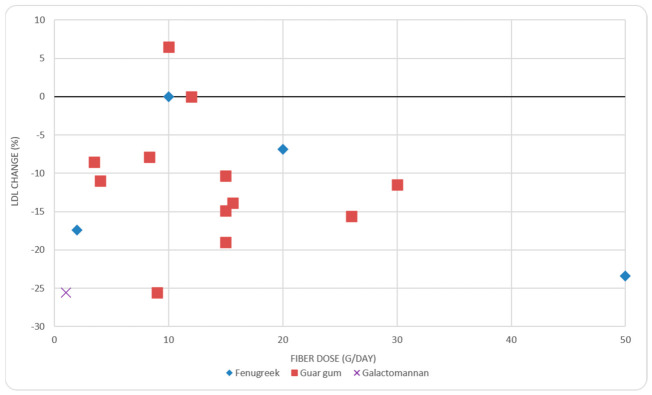
The relationship between fiber dose and the percentage change in LDL cholesterol across the included studies [15,18,20,21,24,29,31,35,37,40,41,44,46,48,56,57,61]. Each point represents an individual study categorized by fiber type.

The magnitude of changes in lipid parameters varied across studies. The largest improvements across all lipid parameters were reported by Geberemeskel et al. following fenugreek supplementation at a dose of 50 g/day for 30 days in patients with type 2 diabetes. Total cholesterol decreased by 29.81 mg/dL, triglycerides decreased by 60.24 mg/dL, and HDL cholesterol increased by 10.5 mg/dL. It is possible that the magnitude of these changes was influenced by the relatively high dose of fenugreek seed powder (50 g/day) administered in this study [24]. All three galactomannan sources demonstrated considerable efficacy in lowering LDL cholesterol levels. However, differences in the intervention doses should be taken into account when interpreting these findings. The relationship between LDL cholesterol reduction and dose is illustrated in Figure 3. The greatest reduction in LDL cholesterol was observed for galactomannan and guar gum, after fenugreek interventions. Study [52], administering 100 g/day, was also excluded from this figure for the reasons described above. In cases where LDL cholesterol levels did not decrease or even showed an increase, the investigators reported that participants had LDL cholesterol concentrations within or close to the normal range at baseline, reducing the likelihood of observing substantial improvements. Furthermore, the duration of the intervention was also cited as a potential factor influencing the limited response.

### 3.5. Body Weight and BMI

Body weight (BW) and body mass index (BMI) were assessed in 31 and 9 of the included studies, respectively. The largest reduction in BW was reported by Deshpande et al., who reported body weight decreases of 5.6% following fenugreek supplementation at a dose of 500 mg/day seed extract [23]. This was followed by Pasman et al. reporting reductions of 4.7% with guar gum at doses of 20 g/day [42]. Additional studies, including Ahmad et al.’s, also demonstrated notable reductions in BW [15]. The greatest decrease in BMI was observed in the study by Cicero et al., in which BMI decreased by 6.5% following guar gum supplementation at a dose of 3.5 g/day [20]. Nevertheless, most studies reported only small changes in BMI and body weight. Overall, both BW and BMI showed modest but relatively consistent reductions across different fiber types and doses.

### 3.6. Risk-of-Bias Assessment

**Figure 4:**
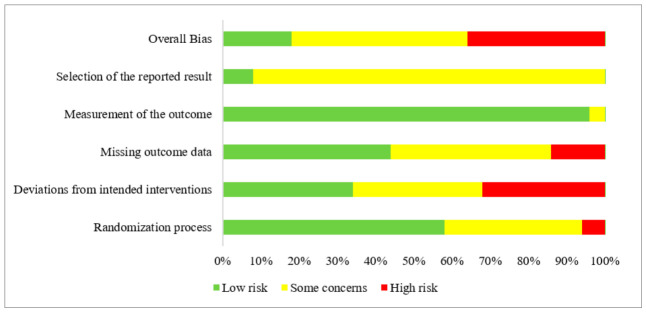
Overall quality of included studies.

Risk-of-bias assessments reporting overall quality of included studies are presented in Figure 4. Overall, most studies were judged to have either some concerns or high risk of bias, with only a minority classified as low risk. The randomization process was generally adequate, with a substantial proportion of studies assessed as low risk, although some concerns remained. Deviations from intended interventions and missing outcome data were among the main contributors to increased risk of bias. A considerable number of studies were rated as having high risk in these domains, reflecting issues such as lack of blinding, co-interventions, and incomplete follow-up. In contrast, measurement of the outcome was consistently assessed as low risk across almost all studies, likely due to the use of objective biochemical markers. However, selection of the reported result was frequently judged as having some concerns, suggesting potential selective reporting.

## 4. Discussion

The aim of this systematic review was to evaluate the effects of galactomannan-type dietary fibers on glycemic, lipid, and anthropometric outcomes and to explore potential differences between galactomannan sources. We analyzed data from a total of 50 randomized controlled trials, including approximately 2100 participants. Our findings indicate that galactomannan-type dietary fibers can modulate glycemic, lipid, and anthropometric outcomes, although the magnitude of these effects appears to vary between different galactomannan sources. 

Based on the available evidence included in this systematic review, fenugreek interventions may provide the most consistent improvements in glycemic parameters, with a higher number of studies reporting statistically significant improvements in any glycemic parameters (*n* = 10) compared to guar gum (*n* = 7) and galactomannan (*n* = 1), while no significant effects were observed for locust bean gum. These results can be explained by the physicochemical and biological properties of fenugreek. It contains several biologically active compounds, including soluble fiber, trigonelline, steroidal saponins such as diosgenin, and 4-hydroxyisoleucine [63]. Among these, the soluble fiber fraction is primarily composed of galactomannans, which are thought to play a key role in the metabolic effects observed. Fenugreek-derived galactomannans are characterized by high viscosity, which can delay gastric emptying and reduce glucose absorption, thereby attenuating both fasting and postprandial glycemic responses [64]. Galactomannans act as prebiotic dietary fibers by selectively promoting the growth of beneficial gut bacteria, including *Roseburia* and *Faecalibacterium*, which are important producers of short-chain fatty acids (SCFAs), particularly butyrate. Increased butyrate production, together with enhanced fecal bile acid excretion, contributes to improved lipid metabolism. The increased loss of bile acids stimulates hepatic conversion of cholesterol into newly synthesized bile acids, thereby reducing circulating LDL cholesterol concentrations [65]. Trigonelline is another major bioactive compound found in fenugreek belonging to the group of alkaloids. It has attracted scientific interest due to its potential antidiabetic, neuroprotective, antioxidant, anti-inflammatory, and lipid-lowering effects. Experimental studies suggest that trigonelline may improve glucose homeostasis and insulin sensitivity, partly through modulation of glucose metabolism, oxidative stress, and inflammatory pathways [66]. 4-hydroxyisoleucine, which may enhance insulin secretion and improve insulin sensitivity, further contributes to its glucose-lowering effects [67]. Diosgenin improves the metabolism of glucose by endorsing the differentiation of adipocytes and hindering inflammation in adipose tissues [68]. In addition, fenugreek saponins are often highlighted as a key component responsible for the lipid-lowering effects [24]. However, in this present review, this effect appeared to be less pronounced, with only three studies reporting statistically significant improvements in lipid parameters.

In contrast, guar gum demonstrated a more substantial effect, with a markedly higher number of studies reporting significant improvements (*n* = 13). One possible explanation for these findings is the higher viscosity-enhancing capacity of guar gum, which may promote more effective binding of bile acids in the intestine compared to lower-viscosity fibers such as fenugreek-derived galactomannans. Increased viscosity slows the movement and absorption of nutrients within the gastrointestinal tract, thereby reducing the interaction between digestive enzymes and dietary substrates, altering intestinal motility, and delaying gastric emptying. Furthermore, viscosity contributes to the thickening of the unstirred water layer adjacent to the intestinal mucosa, which may hinder the diffusion of cholesterol toward the absorptive surface and consequently reduce its intestinal uptake [69]. Regarding body weight and BMI, none of the included studies reported a significant change compared to the control group. However, this does not exclude a potential role of dietary fiber in weight management. Due to their well-documented satiety-enhancing properties, soluble fibers may help reduce overall energy intake by promoting earlier satiation and prolonged fullness [70]. Nevertheless, fiber intake alone is unlikely to lead to clinically significant weight loss unless it is accompanied by changes in other lifestyle factors. Therefore, it should be viewed as part of a more comprehensive lifestyle intervention, within which it can contribute to favorable changes in body weight. This is significant because obesity is one of the most significant global public health challenges, having reached epidemic proportions worldwide. It is estimated that more than one billion people are obese, accounting for approximately 13% of the world’s population [71].

### Strengths and Limitations

This systematic review has several strengths. To our knowledge, it is the first review to comprehensively evaluate and compare the health effects of different galactomannan sources, including guar gum, fenugreek, locust bean gum, and tara gum. By examining these fibers separately rather than treating galactomannans as a homogeneous group, the review provides a more nuanced understanding of their potential physiological and metabolic effects. Furthermore, the inclusion of studies across a range of populations and health outcomes offers a broad overview of the current evidence and highlights potential differences between individual galactomannan sources.

Several limitations should also be acknowledged. First, the available evidence was unevenly distributed across galactomannan types, with the majority of studies focusing on guar gum and fenugreek, whereas considerably fewer investigations were identified for locust bean gum and tara gum. Second, substantial heterogeneity was observed in study populations, intervention characteristics, dosages, treatment durations, and outcome measures, which limited direct comparisons across studies. Third, many of the included studies had relatively small sample sizes and varied methodological quality.

## 5. Conclusions

Our results confirmed substantial differences observed among individual galactomannan sources. Overall, fenugreek-derived interventions appeared to be particularly effective in improving glycemic parameters, whereas guar gum showed more consistent benefits on lipid-related outcomes. Since these fibers can be easily consumed in the form of dietary supplements, it is worth considering their regular, daily intake. Although current recommendations suggest that fiber intake for people with diabetes should be at least 30 g/day, our results show that even a small portion of this amount—e.g., 1 g of galactomannan—can have a beneficial effect on metabolism [72]. This approach may facilitate the practical implementation of daily fiber intake and improve dietary adherence, as it focuses not primarily on increasing the total amount of fiber consumed but rather on improving the quality and functional composition of fiber intake. Furthermore, additional randomized controlled trials are needed to evaluate the effects of fenugreek seed supplementation in women with polyendocrine metabolic ovarian syndrome (PMOS), a population characterized by insulin resistance and dyslipidemia, to determine its potential role in metabolic management.

## Figures and Tables

**Table 1:** Basic characteristics of the included studies.

Study	Population	Participants	Intervention	Control	Dosage	Duration	Outcomes
Abdel-Barry et al., 2000 [13]	Healthy males, aged 20–30 years	20	Fenugreek leaf extract	coffee	40 mg/kg	4 h	FBG, TC
Adam Tanja et al., 2005 [14]	Healthy males and females (normal weight + obese), aged 20–60 years	58	Guar gum	no	2.5 g	2 h	Postprandial glucose, GLP-1, insulin level, satiety
Ahmad et al., 2021 [15]	Males and females with T2DM, age range of 40–65 years	52	Fenugreek seed powder	cinnamon, 4 g	20 g	90 days	FPG, HbA1c, insulin level, TC, LDL, HDL, TG, BW
Aro et al., 1981 [16]	Males and females with T2DM, age range of 39–69 years	9	Guar gum	no	21 g	3 months	FBG, TC, LDL, HDL, TG
Bawadi et al., 2009 [17]	Males and females with T2DM, age range of 40–70 years	166	Fenugreek seeds	dextrose	2.5 g, 5 g	2 h	2 h postprandial glucose
Blake et al., 1997 [18]	Healthy males and females, mean age 44.0 ± 3.6 years	11	Guar gum	no	4 g	6 weeks	TC, LDL, HDL, TG, BW
Chavassus et al., 2009 [19]	Healthy males aged 19–26 years	20	Fenugreek seed extract	no	588 and 1176 mg	3 × 14 days	FBG, TC, TG, BW
Cicetro et al., 2007 [20]	Overweight males and females, aged 50–70 years	141	Guar gum	psyllium	3.5 g	6 months	FBG, HbA1c, TC, LDL, HDL, TG, BMI
Dall”Alba et al., 2013 [21]	Males and females with T2DM, at age >30 years	44	Guar gum	no	10 g	6 weeks	FBG, HbA1c, TC, LDL, HDL, TG, BW
Deshpande et al., 2020 [22]	Females with high fat mass, mean age 27.42 ± 5.38	24	Fenugreek seed extract	placebo	500 mg	8 weeks	BW, BMI
Deshpande et al., 2025 [23]	Males and females with T2DM, aged 42–50 years	29	Fenugreek seed flakes	no	30 g	14 days	FBG, HbA1c, insulin level, BW, BMI
Geberemeskel et al., 2019 [24]	Males and females with T2DM, aged 35–75 years	114	Fenugreek seed powder	no	50 g	30 days	TC, TG, LDL
Hadi et al., 2020 [25]	Males and females with T2DM, aged 35–65 years	48	Fenugreek seed powder	no	15 g	8 weeks	FBG
Hassani et al., 2019 [26]	Males and females with T2DM, aged 35–70 years	64	Fenugreek seed powder	wheat flour	10 g	2 months	FBG, HbA1c, BMI
Hassanzadeh Bashtiana et al., 2013 [27]	Females with PCOS aged 20–35 years	58	Fenugreek seed extract	placebo (lactose)	500 mg	8 weeks	FBG, OGTT glucose, insulin level, HOMA-IR, BW
Heini et al., 1998 [28]	Obese females, average age 46 ± 6 years	25	Guar gum	placebo	20 g	5 weeks	FBG, OGTT glucose, insulin level, BW
Holman et al., 1987 [29]	Males and females with T2DM, aged 31–71 years	29	Guar gum	placebo	15 g	8 weeks	FBG, OGTT glucose, HbA1c, LDL, HDL, TG, BW
Hota et al., 2024 [30]	Males and females with T2DM, average age 53.03 years	204	Fenugreek seed extract	no	1 g	12 weeks	FBG, OGTT glucose, HbA1c, HOMA-IR
Khan et al., 1981 [31]	Healthy males and females, aged 20–50 years	24	Guar gum	placebo	9 g	4 weeks	BW, TC, LDL, TG
Kovacs et al., 2001 [32]	Overweight males, aged 19–56 years	28	Guar gum	no	7.5 g	6 weeks	BW
Kovacs et al., 2002 [33]	Overweight males, average age 44 ± 9 years	15	Guar gum	no	7.5 g	6 weeks	BW
Laajam et al., 1990 [34]	Males and females with T2DM, aged 21–70 years	39	Guar gum	placebo (beef gelatin)	15 g	8 weeks	FBG, HbA1c, OGTT glucose, TC, TG, BW
Lalor et al., 1990 [35]	Males and females with T2DM, aged 40–73 years	26	Guar gum	placebo, metformin	15 g	3 months	BW, FBG, TC, LDL, TG
Lu et al., 2008 [36]	Males and females with T2DM, aged 25–65 years	69	Fenugreek seed extract	placebo	6.3 g	12 weeks	FBG, OGTT glucose, HbA1c, BMI
Luk et al., 2018 [37]	Males and females with pre-diabetes, mean age 56.4 ± 9.1 years	57	Guar gum	placebo	12, 24 g	16 weeks	BW, TC, LDL, HDL, TG
Makkonen et al., 1993 [38]	Menopausal women, mean age 52.3± 2.7 years	30	Guar gum	placebo	15 g	6 months	BW, FBG, TC, TG, HDL
Mathern et al., 2009 [39]	Obese males and females, aged 18–65 years	18	Fenugreek seed extract	no	4, 8 g	3,5 h	Glucose AUC, glucose peak, insulin level AUC, insulin level peak, energy intake, satiety
Mclvor et al., 1986 [40]	Males and females with T2DM, mean age 48.5 ± 2.9 years	16	Guar gum	placebo	26–40 g	6 months	BW, TC, LDL, HDL, TG
Najdi et al., 2019 [41]	Males and females with T2DM, mean age 4.8 ± 2.1 years	9	Fenugreek seeds	glibenclamide	2 g	12 weeks	FBG, HbA1c, HOMA-IR, insulin level, BW, TC, TG, HDL, LDL
Pasman et al., 1997 [42]	Obese females, mean age 41.4 ± 7.4 years	31	Guar gum	no	20 g	14 months	BW, FBG, TC
Pazzi et al., 2021 [43]	Females with fibromyalgia, age > 18 years	64	Locust bean gum	Ganoderma lucidum	6 g	6 weeks	FBG, TG, TC, BW
Peterson et al., 1987 [44]	Males and females with T2DM, aged 47–69 years	16	Guar gum	no	8.3 g	6 weeks	HbA1c, FBG, TC, LDL, TG, BW
Poole et al., 2010 [45]	Resistance-trained males, mean age 21.4 ± 2.8 years	49	Fenugreek seed extract	no	500 mg	8 weeks	BW, TG, TC
Rafraf et al., 2014 [46]	Males and females with T2DM, aged 30–60 years	88	Fenugreek seed powder	placebo	10 g	8 weeks	FBG, HbA1c, insulin level, HOMA-IR, TC, TG, LDL, HDL
Raghuram et al., 1994 [47]	Males and females with T2DM, aged 38–54 years	10	Fenugreek seed powder	no	25 g	15 days	BW
Rashid et al., 2019 [48]	Males and females with T2DM, aged 30–60 years	64	Extracted galactomannan	placebo	1 g	12 weeks	FBG, HbA1c, TG, TC, LDL, HDL, BMI
Rehman et al., 2021 [49]	Males and females with T2DM, aged 30–65 years	60	Fenugreek seed powder	no	20 g	2 months	FBG, HbA1c, BMI
Requejo et al., 1990 [50]	Males and females with T2DM, aged 41–72 years	12	Guar gum	placebo	15 g	4 weeks	FBG, TC, TG, BW
Robert et al., 2016 [51]	Healthy males and females, aged 21–48 years	10	Fenugreek seed powder	no	8–9 g	2 h	Glucose AUC, glycemic index
Sharma et al., 1990 [52]	Males and females with T2DM, aged 35–58 years	15	Fenugreek seed powder	no	100 g	20 days	FBG, TC, LDL, TG, HDL
Superko et al., 1988 [53]	Males with elevated plasma cholesterol, mean age 51 ± 12 years	50	Guar gum	no	15 g	8 weeks	TC, LDL, TG, HDL
Trask et al., 2014 [54]	Males and females with T2DM, mean (SD) age 59 (9.8) years	24	Galactomannan derivative	no	8, 16 g	2 h	2 h postprandial glucose excursion
Tredger et al., 1991 [55]	Healthy males, aged 24–56 years	15	Guar gum	sugar beet fiber, wheat bran	20 g	14 days	TC, HDL, TG, FBG, insulin level
Tuomilehto et al., 1988 [56]	Males and females with hypercholesterolemia, mean age 56.9 years	23	Guar gum	no	15–30 g	1 year	TC, LDL, TG, HDL, BW
Turner et al., 1990 [57]	Males and females with primary hyperlipidemia, mean age 62 years	9	Guar gum	no	30 g	6 weeks	TC, LDL, IDL, TG, HDL, BW
Uusitupa et al., 1989 [58]	Males and females with T2DM, mean age of males 58.6 ± 5.4 years and of females 61.4 ± 6.3 years	39	Guar gum	no	15 g	3 months	FBG, HbA1c, TC, HDL, TG, BW
Vaaler et al., 1986 [59]	Males and females with T1DM, mean age 27 years	28	Guar gum	bran	29 g	3 months	FBG, postprandial glucose, HbA1c, TC, TG, BW
Van Duyn et al., 1986 [60]	Males and females with T2DM, mean age 49 ± 1.92 years	16	Guar gum	placebo	30 g	6 months	BW, energy intake, carbohydrate intake, fat intake, protein intake, nutritional status
Wirth et al., 1982 [61]	Males and females with hypercholesterolemia, mean age 51.7 years	12	Guar gum	no	15.6 g	3 months	TC, LDL, HDL, TG, BW, FBG
Wolf et al., 2003 [62]	Healthy males and females, mean age 51 ± 3 years	30	Guar gum	placebo	5 g	2 h	Glucose AUC, peak glucose, relative glycemic response, satiety, gastrointestinal tolerance

T2DM: type 2 diabetes; PCOS: polycystic ovary syndrome; FBG: fasting blood glucose; FI: fasting insulin; TG: triglyceride; TC: cholesterol; HDL: high-density lipoprotein cholesterol; LDL: low-density lipoprotein cholesterol; OGTT: Oral Glucose Tolerance Test; AUC: area under the curve; BW: body weight; BMI: body mass index.

## Data Availability

No new data were created in this study. Data sharing is not applicable to this article.

## Abbreviations

The following abbreviations are used in this manuscript:

T2DM	Type 2 diabetes
PCOS	Polycystic ovary syndrome
PMOS	Polyendocrine metabolic ovarian syndrome
FBG	Fasting blood glucose
FI	Fasting insulin
TG	Triglyceride
TC	Total cholesterol
HDL	High-density lipoprotein cholesterol
LDL	Low-density lipoprotein cholesterol
OGTT	Oral Glucose Tolerance Test
AUC	Area Under the Curve
BW	Body weight
BMI	Body Mass Index
IBD	Inflammatory Bowel Disease
GERD	Gastroesophageal reflux disease
RCTs	Randomized controlled trial
RoB 2	Risk-of-bias tool for randomized trials
SCFAs	Short-chain fatty acids
